# Oncostatin M receptor regulates osteoblast differentiation via extracellular signal-regulated kinase/autophagy signaling

**DOI:** 10.1186/s13287-022-02958-1

**Published:** 2022-06-28

**Authors:** Jie Zhou, Junying Yang, Yuan Dong, Yaru Shi, Endong Zhu, Hairui Yuan, Xiaoxia Li, Baoli Wang

**Affiliations:** 1 NHC Key Lab of Hormones and Development, Tianjin Key Lab of Metabolic Diseases, Chu Hsien-I Memorial Hospital and Institute of Endocrinology, Tianjin Medical University, 6 Huan-Rui-Bei Road, Tianjin, 300134 China; 2 College of Basic Medical Sciences, Tianjin Medical University, 22 Qi-Xiang-Tai Road, Tianjin, 300070 China

**Keywords:** Autophagy, Osteoblast, Differentiation, Bone homeostasis, Oncostatin M receptor, Extracellular signal-regulated kinase

## Abstract

**Background:**

Oncostatin M receptor (OSMR), as one of the receptors for oncostatin M (OSM), has previously been shown to mediate the stimulatory role of OSM in osteoclastogenesis and bone resorption. However, it remains to be clarified whether and how OSMR affects the differentiation of osteoblasts.

**Methods:**

The expression level of OSMR during osteoblast and adipocyte differentiation was examined. The role of OSMR in the differentiation was investigated using in vitro gain-of-function and loss-of-function experiments. The mechanisms by which OSMR regulates bone cell differentiation were explored. Finally, in vivo function of OSMR in cell fate determination and bone homeostasis was studied after transplantation of OSMR-silenced bone marrow stromal cells (BMSCs) to the marrow of ovariectomized mice.

**Results:**

OSMR was regulated during osteogenic and adipogenic differentiation of marrow stromal progenitor cells and increased in the metaphysis of ovariectomized mice. OSMR suppressed osteogenic differentiation and stimulated adipogenic differentiation of progenitor cells. Mechanistic investigations showed that OSMR inhibited extracellular signal-regulated kinase (ERK) and autophagy signaling. The downregulation of autophagy, which was mediated by ERK inhibition, suppressed osteogenic differentiation of progenitor cells. Additionally, inactivation of ERK/autophagy signaling attenuated the stimulation of osteogenic differentiation induced by Osmr siRNA. Furthermore, transplantation of BMSCs in which OSMR was silenced to the marrow of mice promoted osteoblast differentiation, attenuated fat accumulation and osteoclast differentiation, and thereby relieved the osteopenic phenotype in the ovariectomized mice.

**Conclusions:**

Our study has for the first time established the direct role of OSMR in regulating osteogenic differentiation of marrow stromal progenitor cells through ERK-mediated autophagy signaling. OSMR thus contributes to bone homeostasis through dual regulation of osteoblasts and osteoclasts. It also suggests that OSMR may be a potential target for the treatment of metabolic disorders such as osteoporosis.

**Supplementary Information:**

The online version contains supplementary material available at 10.1186/s13287-022-02958-1.

## Background

Osteoporosis, especially that in aging or postmenopausal patients, is characterized by imbalanced osteoblastic bone formation and osteoclastic bone resorption and increased marrow fat accumulation [1, 2]. Osteoblasts and adipocytes are both derived from bone marrow stromal cells (BMSCs) and a reciprocal and inverse relationship exists between adipogenesis and osteogenesis [3]. It is widely accepted that the aberrant lineage commitment of endogenous BMSCs in favor of adipogenesis contributes to the development of osteoporosis [4].

Osteogenic and adipogenic differentiation of BMSCs are regulated by a variety of transcription factors and signaling pathways, including runt-related transcription factor 2 (Runx2), osterix (Osx), peroxisome proliferator-activated receptor γ (PPARγ), CCAAT/enhancer-binding proteins (C/EBPs), bone morphogenetic proteins (BMPs), Wnt/β-catenin, Hedgehog, and extracellular signal-regulated kinase (ERK) pathway [5–10].

As a highly conserved catabolic process in eukaryotic cells, autophagy consists in the sequestration of damaged organelles or protein aggregates in double-walled vesicles, followed by the delivery to lysosome/vacuole, where the cargos are degraded and recycled [11]. Autophagy is recognized as a mechanism to maintain cellular homeostasis and protect against stress and inflammatory conditions, aging, and cancer. Many efforts have been made to elucidate the relationship between autophagy and bone homeostasis [12–15]. Emerging evidence has shed light on the role of autophagy in lineage determination of MSCs, with deficiency of autophagy reducing osteoblast differentiation and mineralization [12, 16, 17].

Oncostatin M receptor (OSMR) is a member of the interleukin 6 (IL-6) receptor family. It is capable of transducing oncostatin M (OSM)-induced signaling events when heterodimerizing with gp130 to form the high affinity receptor for OSM [18]. Besides, OSMR also associates with interleukin 31 (IL-31) receptor A to form the IL-31 receptor and thus transduces IL-31-induced signaling events [18]. The expression of OSMR has been detected in a variety of cell types including osteoblasts and osteocytes [19–21].

Upon OSM binding, OSMR plays key roles in a variety of physiological and pathophysiological processes, including hematopoiesis [22], lung fibrosis [23], tumorigenesis [24], rheumatoid arthritis [25], and bone homeostasis [21, 26, 27]. Previous studies demonstrated that OSMR is able to stimulate osteoclast differentiation in a RANKL-dependent manner [21, 28]. To date, it remains to be clarified if and how OSMR regulates osteogenic differentiation of progenitor cells.

In the current study, using in vitro and in vivo experiments, we have for the first time investigated the direct role of OSMR in osteogenic differentiation of marrow stromal progenitor cells and elucidated the mechanism for the control of osteogenic differentiation by OSMR.

## Methods

### Osteoblast and adipocyte differentiation

BMSCs were flushed with a-MEM from femurs and tibiae of 4-week-old C57BL/6J mice, seeded in 25 cm^2^ flasks as previously reported [29], and cultured in a-MEM containing 10% fetal bovine serum (FBS). Stromal line ST2 cells were maintained in DMEM containing 10% FBS.

To induce osteogenic differentiation, 80% confluent cells were cultured in osteogenic medium (a-MEM containing 10% FBS, 50 μg/ml ascorbic acid, and 5 mM β-glycerophosphate) for 14 d followed by ALP staining, or for 21 d followed by alizarin red staining. To induce adipogenic differentiation, confluent cells were cultured for 72 h in adipogenic medium (a-MEM containing 10% FBS, 5 μg/ml insulin, 0.5 μM dexamethasone, 0.25 mM methylisobutylxanthine, and 50 μM indomethacin). Subsequently, cells were switched to a-MEM containing 10% FBS and 5 μg/ml insulin for additional 48–72 h followed by oil-red O staining.

### Quantitative RT‑PCR

Total RNA was isolated from tissues using RNAiso plus reagent (TaKaRa, Dalian, China), or from cultured cells using a RNA isolation kit (GMbiolab, Taiwan). Reverse transcription was performed using 1 μg total RNA and RevertAid First-Strand cDNA Synthesis Kit (Thermo Fisher Scientific, Waltham, MA, USA). Amplification reactions were carried out using SYBR Green PCR Master Mix (Sangon Biotech, Shanghai, China). Primer sequences are listed in Additional file 1: Table S1. The expression levels of the target genes were normalized against that of β-actin and analyzed using the ΔΔCt method.

### Lentiviral packaging and infection

The plasmid harboring Osmr shRNA sequence was constructed according to previously described protocol [30]. The silencing lentivirus (Osmr-KD LV) was packaged in 293T cells with the lentiviral packaging system (Jiman Biotech, Shanghai, China). The lentivirus packaged with the empty vector was used as control. Primary BMSCs were infected with the viruses with a multiplicity of infection (MOI) of 20.

### Constructs and transfection

The translated region of Osmr cDNA was PCR-amplified and subsequently subcloned into pcDNA3.1 vector using the ClonExpress II One Step Cloning Kit (Vazyme, Nanjing, China).

In Osmr gain-of-function experiments, ST2 cells were transfected with Osmr expression construct or empty vector by using Attractene Transfection Reagent (QIAGEN, German) for 16 h. In Osmr loss-of-function experiments, cells were transfected with 20 nM siRNAs or control siRNA (GenePharma, Shanghai, China) by using Lipofectamine RNAiMax (Life Technologies, Gaithersburg, MD, USA) for 20 h. Subsequently, ST2 cells were cultured in adipogenic or osteogenic medium at appropriate confluence. The siRNA sequences used are listed in Additional file 1: Table S2.

### Cell viability assay

ST2 cells were seeded in 96-well plates at a density of 1.5 × 10^4^ cells per well and transfected with constructs or siRNAs at appropriate confluence. Twenty-four hours after transfection, the effect of OSMR on viability was determined by using a CCK-8 assay kit (Dojindo, Kumamoto, Japan).

### Cell proliferation assay

5-ethynyl-2′-deoxyuridine (EdU) staining was performed using BeyoClick™ EdU Cell Proliferation Kit with Alexa Fluor 594 (Beyotime, Shanghai, China). Briefly, ST2 cells or BMSCs were incubated with 10 μM EdU for 2 h followed by fixation in 4% paraformaldehyde for 15 min. Subsequently, the cells were permeabilized with 0.3% Triton X-100 for 15 min and then incubated in the dark with Click Additive Solution for 30 min. Cell nuclei were stained with Hoechst 33342 for 10 min. The EdU-positive cells were determined using laser scanning confocal microscope.

### Oil-red O staining

Lipid droplet formation in differentiated adipocytes was detected by staining with oil-red O [31]. Differentiated adipocytes were fixed in 4% paraformaldehyde for 10 min and then stained with 0.3% oil-red O solution in 60% saturated isopropanol for 5 min. To quantify the retention of oil-red O, the stain was extracted using isopropanol and absorbance was measured by spectrophotometry at 520 nm.

### Alkaline phosphatase (ALP) staining and alizarin red staining

ALP staining was carried out after 14 days of osteogenic treatment. Briefly, cells were fixed in 4% paraformaldehyde for 10 min and stained for 15 min using a NBT/BCIP staining kit (Beyotime, Shanghai, China). Alizarin red staining was performed after 21 days of osteogenic treatment. Briefly, after fixing for 10 min with 4% paraformaldehyde, the cells were stained for 15 min with 1% alizarin red staining solution (pH 4.2).

### Western blotting

Cells were lysed by RIPA buffer supplemented with protease inhibitor. The proteins were separated by SDS-PAGE and transferred onto nitrocellulose membranes. The membranes were incubated with primary antibodies, then with horseradish peroxidase (HRP)-conjugated secondary antibodies, and visualized by chemiluminescence reagent (Proteintech, Wuhan, Shanghai). The primary antibodies used included antibodies produced by Abcam (Cambridge, MA, USA): anti-osterix, anti-ALP, and anti-osteopontin (OPN); antibodies by Cell Signaling Technology (Danvers, MA, USA): anti-PPARγ, anti-C/EBPα, anti-phospho-src homology 2 domain-containing transforming protein C1 (SHC1) (Tyr317), anti-ERK1/2, and anti-phospho-ERK1/2 (Thr202/Tyr204); antibody by ABclonal (Wuhan, China): anti-nuclear factor of activated T cells 1 (NFATC1); antibody by Beyotime (Shanghai, China): anti-cathepsin K (CTSK); antibodies by Proteintech (Wuhan, China): anti-OSMR, anti-fatty acid-binding protein 4 (FABP4), anti-microtubule-associated protein 1 light chain 3 (LC3), anti-autophagy-related gene 5 (ATG5), anti-autophagy-related gene 7 (ATG7), anti-Beclin 1, anti-P62, anti-SHC1, and anti-β-actin. The expression levels of the target genes were analyzed by dividing the grayscale of the target bands with that of β-actin.

### In vitro osteoclast differentiation

Bone marrow cells were collected from the tibiae and femurs of 8-week-old mice and seeded on 48-well plates at the density of 3 × 10^5^/well. The cells were cultured for 3 days in a-MEM containing 10% FBS and 20 ng/ml macrophage colony-stimulating factor (M-CSF) to facilitate the growth of monocyte/macrophage precursor cells. On the other hand, primary BMSCs isolated from 4- to 6-week-old mice were cultured and infected with Osmr shRNA lentivirus or control virus at passages 3–5. The BMSCs were then added to the monocyte/macrophage precursor cultures at the density of 2000/well and cocultured in the presence of 20 ng/ml M-CSF and 10^−7^ mol/l 1,25(OH)_2_ VitD_3_ for osteoclast generation. Culture medium was changed every 3 days. After 5 days of induction, total RNA and protein were extracted and qRT-PCR and Western blotting were performed. After 7 days of induction when large multinucleated cells were observed under microscope, the cultures were fixed with 4% paraformaldehyde and then stained for tartrate-resistant acid phosphatase (TRAP) activity using a TRAP staining kit (Sigma-Aldrich, St. Louis, MO, USA) following the manufacturer's protocol. TRAP-positive multinucleated osteoclasts with ≥ 3 nuclei were counted under a light microscope.

### Bone marrow cavity transplantation of BMSCs

C57BL/6J mice were purchased from Si Pei Fu Biological Technology (Beijing, China). Ten-week-old female mice were randomly divided into four groups (17 mice in each group), i.e., “Sham/Ctrl LV” group, “Sham/Osmr-KD LV” group, “OVX/Ctrl LV” group, and “OVX/Osmr-KD LV” group. The four groups received either sham operation or bilateral ovariectomy (OVX) surgery.

Primary BMSCs were obtained from the tibiae and femurs of 6-week-old mice and cultured in a-MEM containing 10% FBS. The cells were infected with Osmr-KD LV or control LV for 24 h. Three days after surgery, the transplantation of the BMSCs was carried out with reference to previous report [32]. Briefly, the knee of the mouse was flexed to 90 degree. 2.5 × 10^5^ BMSCs suspended in Hank's Balanced Salt Solution (HBSS) were injected using a microsyringe, whose needle was inserted into the joint surface of the tibia through the patellar tendon and then into the bone marrow cavity. Mice were transplanted once a month and killed 2 weeks, 4 weeks, or 12 weeks after surgery. Two weeks after surgery, bone marrow cells were isolated from the transplanted tibiae and osteoclast formation assay was performed. The transplanted tibiae were dissected and subjected to histological and immunohistochemical staining 4 weeks after surgery, or μCT analysis 12 weeks after surgery.

All mice were housed under specific pathogen-free conditions with food and water easily accessible. The animal experiments were performed in accordance with the Chinese guidelines for animal welfare and experimental protocol and was approved by the Animal Ethics Committee of Tianjin Medical University Chu Hsien-I Memorial Hospital.

### Ex vivo osteoclast differentiation

Bone marrow cells were collected from the transplanted tibiae of the mice 2 weeks after surgery and then seeded onto 48-well plates at the density of 3 × 10^5^/well in a-MEM supplemented with 10% FBS and 20 ng/ml M-CSF. After 3 days of culturing, the nonadherent cells were discarded and the adherent cells were cultured in the presence of 20 ng/ml M-CSF and 10^–7^ mol/l 1,25(OH)_2_VitD_3_. After 5 days of induction, total RNA and protein were extracted and qRT-PCR and Western blotting were performed. After 7 days of induction, the differentiated osteoclasts were fixed with 4% paraformaldehyde and then subjected to TRAP staining. The TRAP-positive multinucleated osteoclasts were counted.

### Micro-computed tomography (μCT) analysis

Tibia tissues were detached after killing of mice and fixed in 4% paraformaldehyde, then scanned and analyzed by using VivaCT 80 Scanner μCT (Scanco Medical, Switzerland) with a resolution of 10 μm. One-mm-long area starting from 0.1 mm below the growth plate in the proximal tibia was chosen as the region of interest for trabecular bone analysis. The three-dimensional structures were reconstructed, and histomorphometric parameters were analyzed.

### Histological analysis and immunohistochemical staining

Tibia tissues dissected from the mice were fixed with 10% neutral buffered formalin (NBF) for 72 h followed by decalcification in 14% EDTA (pH 7.4) for 3 weeks. Tissues were embedded in paraffin, and 5-μm sagittal-oriented sections were prepared for histological analyses with hematoxylin–eosin (H&E) staining and TRAP staining. Subsequently, osteoblast number, osteoclast number and surface, and adipocyte number and area were measured and calculated, respectively.

Immunohistochemical staining was performed as previously described [33], using primary anti-ALP antibody (Abcam, Cambridge, MA) at 4 °C overnight. Subsequently, an HRP-DAB system (Proteintech, Wuhan, China) was used to detect the immunoreactivity, followed by counterstaining with hematoxylin. The region of interest selected for histological and immunohistochemical staining analysis is 1 mm in length from 0.1 mm below the growth plate in proximal tibial metaphysis.

### Statistical analysis

Data are expressed as mean ± SD. For the quantifications of mRNA and proteins, the means of the control groups were set to 1. Statistical analysis was performed with independent t test for the comparisons between two groups or with ANOVA for those among multiple groups. One-way ANOVA was performed when there was one independent variable, and two-way ANOVA was done when there were two independent variables. If ANOVA indicated significant difference, a post hoc comparison was performed with Tukey’s test. *p* < 0.05 is considered significantly different.

## Results

### Osmr was regulated during osteogenic and adipogenic differentiation

The expression of Osmr was detected in various tissues of 8-week-old C57BL/6J mice, and the results showed that Osmr was affluent in heart, perirenal white fat, skeletal muscle, brown fat, bone, and lung (Additional file 1: Fig. S1A).

Then, we demonstrated by using qRT-PCR that Osmr level increased at day 3 through 13 and peaked at day 9 during osteogenic differentiation of BMSCs (Additional file 1: Fig. S1B). By contrast, it increased during the early stage of adipogenic differentiation of BMSCs, peaked at day 2, and decreased to a level below the baseline (day 0) at day 5 after adipogenic treatment (Additional file 1: Fig. S1C). In addition, Osmr level was significantly increased in the radial metaphysis of ovariectomized mice as compared to that in sham-operated mice (Additional file 1: Fig. S1D). Moreover, it was higher in the radius of 18-month-old mice than in that of 3-month-old mice (Additional file 1: Fig. S1E).

### Silencing of OSMR in progenitor cells enhanced osteogenesis and inhibited adipogenesis

**Fig. 1:**
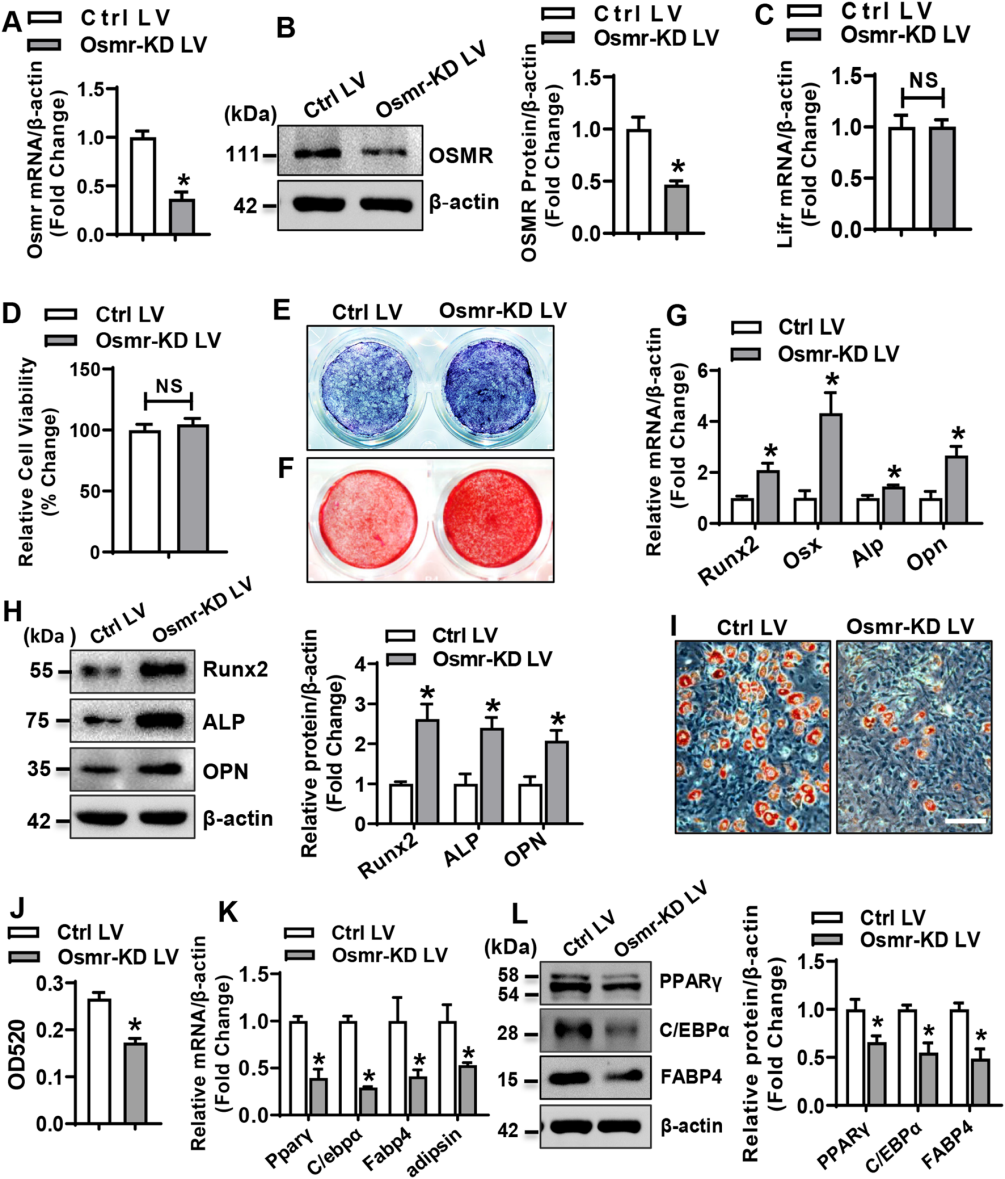
Silencing of OSMR in BMSCs enhanced osteogenesis and inhibited adipogenesis. Silencing of OSMR in BMSCs after the infection of control or Osmr-KD lentivirus was detected using qRT-PCR (**A**) and Western blotting (**B**). The mRNA level of Lifr was detected using qRT-PCR (**C**). The effect of OSMR silencing on the viability of BMSCs was detected by CCK-8 assay (**D**). ALP staining (**E**) and alizarin red staining (**F**) of differentiated osteoblasts were done. The mRNA (**G**) and protein (**H**) levels of osteogenic factors were examined. Lipid droplet formation in differentiated adipocytes was detected by staining with oil-red O (**I**). Oil-red O stain was extracted and absorbance was measured by spectrophotometry at 520 nm (**J**). The mRNA (**K**) and protein (**L**) levels of adipogenic factors were examined. Scale bar in (**I**): 50 μm. Values are mean ± SD, *n* = 3 in (**A**–**C**, **G**, **H**, **J**–**L**), *n* = 9 in (**D**). *p < 0.05 *vs.* Control LV

The role of OSMR in the differentiation of primary BMSCs was investigated. The efficacy of silencing lentivirus (Osmr-KD LV) in knocking down Osmr was verified in primary BMSCs at either mRNA level or protein level (Fig. 1A, B). The silencing of Osmr did not change the expression of leukemia inhibitory factor receptor (LIFR) (Fig. 1C). There was no significant change in cell viability (Fig. 1D) and proliferation (Additional file 1: Fig. S2A, B) of BMSCs following Osmr silencing, evaluated by using CCK-8 assay and 5-ethynyl-2′-deoxyuridine (EdU) staining, respectively. After osteogenic treatment, silencing of Osmr potentiated osteogenesis of BMSCs, as revealed by enhanced ALP staining at day 14 and alizarin red staining at day 21 as compared to the control (Fig. 1E, F). Accordingly, knockdown of Osmr increased the mRNA and protein levels of the key factors for osteogenesis (mRNAs: increased by 1.5–4.3-fold; proteins: increased by 2.1–2.6-fold), including Runx2, osterix, ALP, and osteopontin (OPN) in the cells 72 h after osteogenic treatment (Fig. 1G, H).

By contrast, in the presence of adipogenic medium, the formation of adipocytes was significantly alleviated in Osmr-silenced BMSCs, as evidenced by the decrease in the intensity of oil-red O staining (Fig. 1I, J). Consistently, the mRNA and protein levels of the key factors for adipogenesis including PPARγ, C/EBPα, FABP4, and adipsin were significantly decreased (mRNAs: decreased by 47–71%; proteins: decreased by 34–51%) in Osmr-silenced BMSCs 48 h and 72 h, respectively, after adipogenic treatment (Fig. 1K, L).

Additionally, we also investigated the role of OSMR in the differentiation of ST2 stromal cells. The two independent siRNAs targeting different regions of Osmr were proved to work well in ST2 cells by using qRT-PCR and Western blotting (Additional file 1: Fig. S3A, B). There was no significant change in the viability (Additional file 1: Fig. S3C) and proliferation (Additional file 1: Fig. S4A, B) of ST2 cells following Osmr silencing, evaluated by using CCK-8 assay and EdU staining, respectively. After osteogenic induction, Osmr siRNAs potentiated osteogenesis of ST2, as revealed by enhanced ALP staining (Additional file 1: Fig. S3D) and alizarin red staining (Additional file 1: Fig. S3E), and the increased mRNA/protein levels of osteogenic factors (Additional file 1: Fig. S3F, G). By contrast, silencing of Osmr in ST2 cells suppressed adipogenic differentiation and the expression of adipogenic factors (Additional file 1: Fig. S5A-D).

### Enforced expression of OSMR in progenitor cells suppressed osteogenesis and promoted adipogenesis

**Fig. 2:**
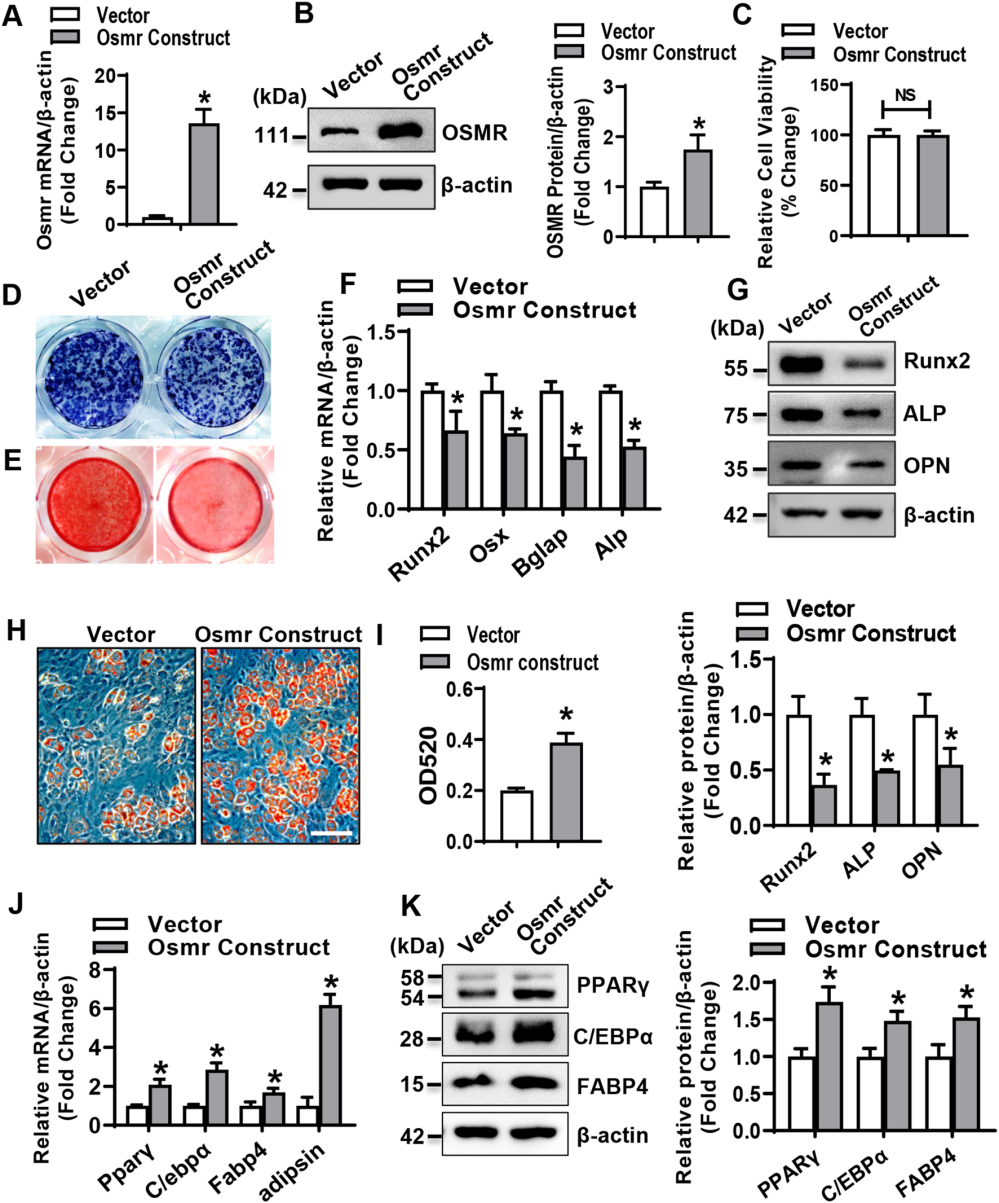
Enforced expression of OSMR in progenitor cells suppressed osteogenesis and promoted adipogenesis. Overexpression of OSMR in ST2 was verified using qRT-PCR (**A**) and Western blotting (**B**). The effect of OSMR overexpression on the viability of ST2 cells was detected by CCK-8 assay (**C**). ALP staining (**D**) and alizarin red staining (**E**) of differentiated osteoblasts were done. The mRNA (**F**) and protein (**G**) levels of osteogenic factors were examined. Lipid droplet formation in differentiated adipocytes was detected by staining with oil-red O (**H**). Oil-red O dye was extracted and absorbance was measured by spectrophotometry at 520 nm (**I**). The mRNA (**J**) and protein (**K**) levels of adipogenic factors were examined. Scale bar in (**H**): 50 μm. Values are mean ± SD. **A**, **B**, **F**, **G**, **I**–**K**, *n* = 3. **C**, *n* = 6. *p < 0.05 *vs.* Vector

Overexpression of OSMR in ST2 cells after transfection of Osmr expression construct was verified with qRT-PCR and Western blotting (Fig. 2A, B). CCK-8 assay and EdU staining showed that an increase in Osmr level in ST2 cells had no effect on cell viability (Fig. 2C) and proliferation (Additional file 1: Fig. S6A, B). In the presence of osteogenic induction, Osmr overexpression blunted osteogenesis, as revealed by reduced ALP staining and alizarin red staining of differentiated osteoblasts at day 14 and day 21, respectively, as compared to vector transfection (Fig. 2D, E). Accordingly, Osmr overexpression decreased the mRNA/protein levels of osteogenic factors 72 h after osteogenic treatment (Fig. 2F, G).

By contrast, Osmr overexpression stimulated the formation of adipocytes from ST2 cells, as evidenced by the dramatic increase in the intensity of oil-red O staining as compared to vector transfection (Fig. 2H, I). Consistently, the mRNA and protein levels of the adipogenic factors were higher in Osmr-overexpressing cells than in control cells 48 h and 72 h, respectively, after adipogenic treatment (Fig. 2J, K).

### OSMR inactivated ERK signaling pathway and suppressed autophagy

**Fig. 3:**
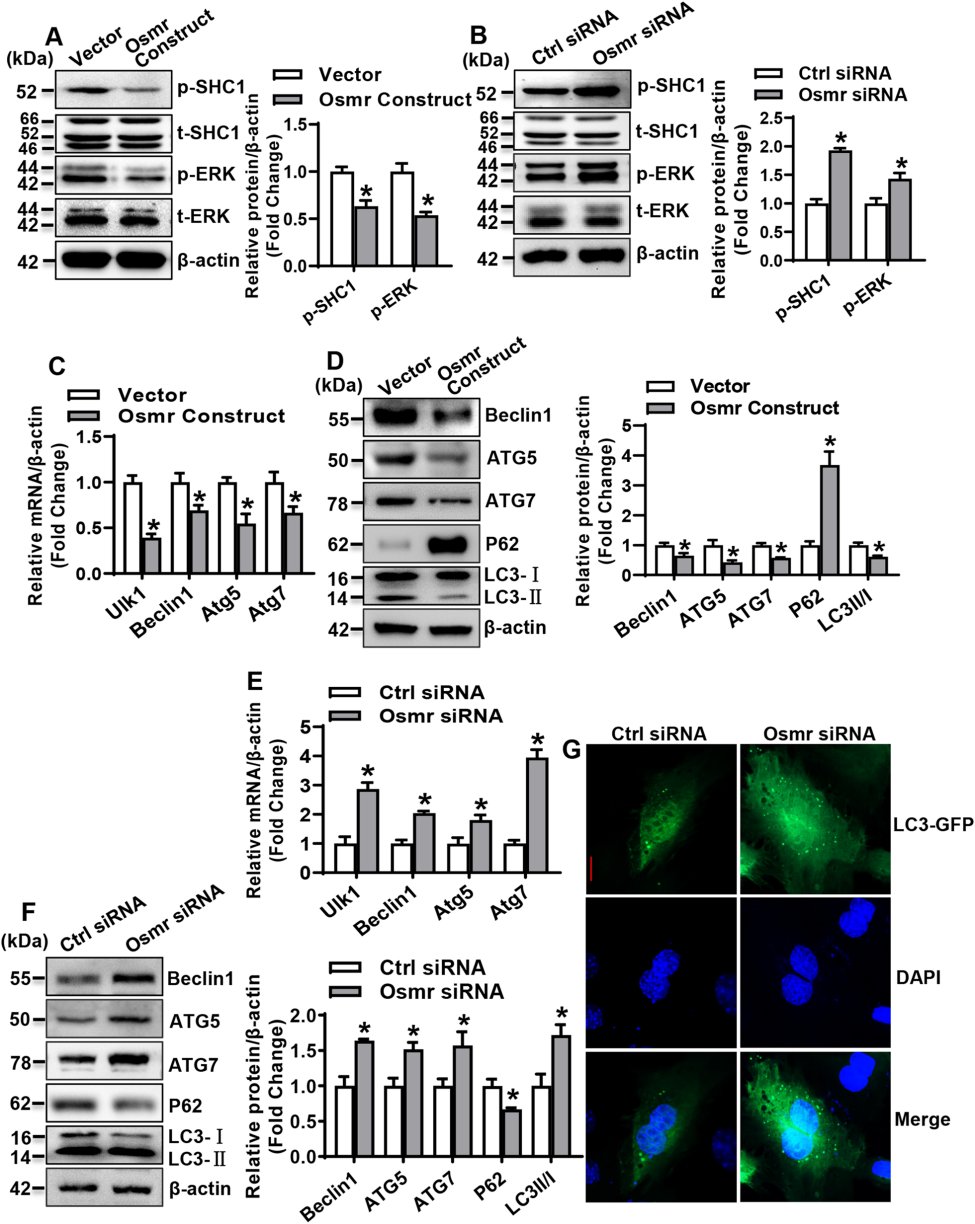
OSMR inactivated ERK signaling pathway and suppressed autophagy. Levels of SHC1 and ERK1/2 in ST2 were examined 48 h after OSMR overexpression (**A**) or silencing (**B**) by Western blotting. The mRNA (**C**: overexpression; **E**: silencing) and protein (**D**: overexpression; **F**: silencing) levels of the key factors for autophagy were examined 48 h after transfection. The level of LC3 puncta formation was analyzed in ST2 cells infected with GFP-LC3 adenovirus for 24 h followed by transfection with Osmr siRNA or control siRNA for 24 h. Representative confocal microscopic pictures are shown (**G**). Scale bar: 10 μm. Values are mean ± SD (*n* = 3). *p < 0.05 *vs.* Vector or Ctrl siRNA

We further explored the mechanism for the regulation of directional differentiation of stromal progenitor cells by OSMR. The levels of SHC1 and ERK1/2 after overexpression or silencing of Osmr were investigated. The results showed that the phosphorylated proteins of SHC1 and ERK1/2 were significantly decreased in Osmr-overexpressing ST2 cells, while they increased in Osmr-silenced cells (Fig. 3A, B).

As ERK signaling is a known activator of autophagy [34, 35], we further explored whether OSMR regulates autophagy. The mRNA and/or protein levels of the key molecules of autophagy, such as Unc-51 like kinase 1 (ULK1), Beclin1, ATG5, and ATG7, and the ratio of LC3II/LC3I were significantly decreased, while the level of P62 was remarkably increased in ST2 cells following Osmr overexpression (Fig. 3C, D). Conversely, after the knockdown of Osmr, the mRNA and/or protein levels of ULK1, Beclin1, ATG5, ATG7, and LC3II/LC3I were increased, while the level of P62 was reduced (Fig. 3E, F). Additionally, the level of LC3 puncta formation, which reflects the transient autophagosomal content based on the balance between the generation and degradation of autophagosomes [36], was analyzed in ST2 cells infected with GFP-LC3 adenovirus. We found that in the cells transfected with Osmr siRNA, the LC3 puncta formation was enhanced (Fig. 3G).

### ERK signaling-mediated autophagy in stromal progenitor cells

**Fig. 4:**
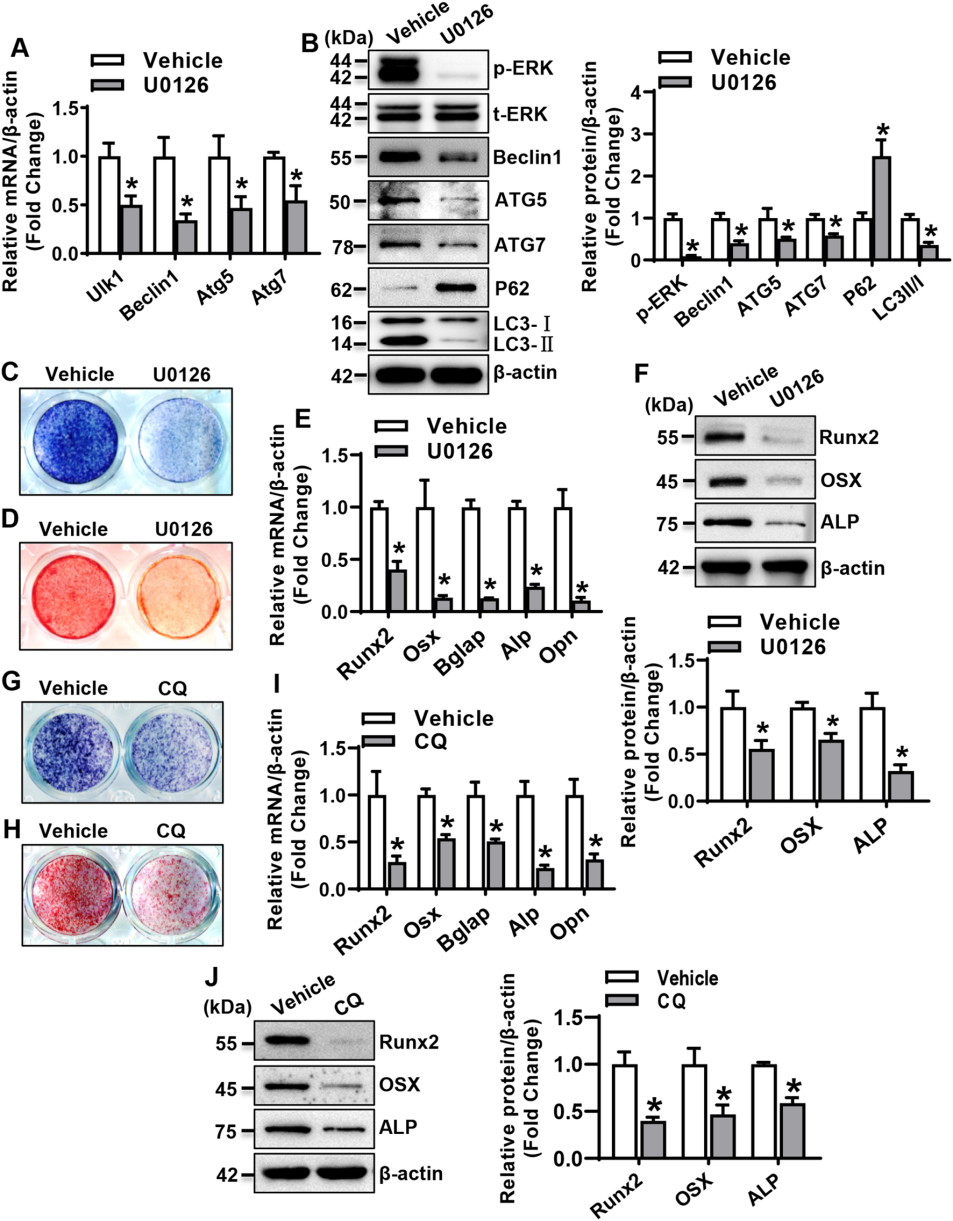
ERK signaling-mediated autophagy in stromal progenitor cells. The mRNA (**A**) and protein (**B**) levels of the key factors for autophagy in ST2 were examined 48 h after U0126 treatment (20 μM). ST2 cells were treated with 20 μM U0126 or chloroquine (CQ) for 24 h, followed by osteogenic treatment. ALP staining (**C**, **G**) and alizarin red staining (**D**, **H**) of differentiated osteoblasts from ST2 were done. The mRNA (**E**, **I**) and protein (**F**, **J**) levels of osteogenic factors were detected 72 h after osteogenic treatment. Values are mean ± SD (*n* = 3). *p < 0.05 *vs.* vehicle treatment

U0126, the inhibitor of ERK signaling, was used to further analyze the effect of ERK signaling on autophagy and osteogenic differentiation. As expected, the phosphorylated protein level of ERK1/2 was downregulated in ST2 cells treated with U0126 (Fig. 4A). Moreover, the autophagy signaling was downregulated following the inactivation of ERK, as evidenced by the decreased levels of Beclin1, ATG5, ATG7, and LC3II/LC3I, and the increased level of P62 (Fig. 4B). Furthermore, in the presence of osteogenic medium, U0126 treatment blunted ALP staining and alizarin red staining of differentiated osteoblasts (Fig. 4C, D). Accordingly, the mRNA and protein levels of the key osteogenic factors were significantly declined (Fig. 4E, F). In addition, chloroquine (CQ), the autophagic pharmacological inhibitor, was also used to analyze the effect of autophagy on osteogenic differentiation. ALP staining and alizarin red staining of differentiated osteoblasts were attenuated after autophagy inactivation with chloroquine treatment (Fig. 4G, H). The mRNA and protein levels of the key osteogenic factors were significantly reduced in the cells treated with chloroquine (Fig. 4I, J).

### OSMR-regulated osteogenic differentiation via ERK/autophagy signaling

**Fig. 5:**
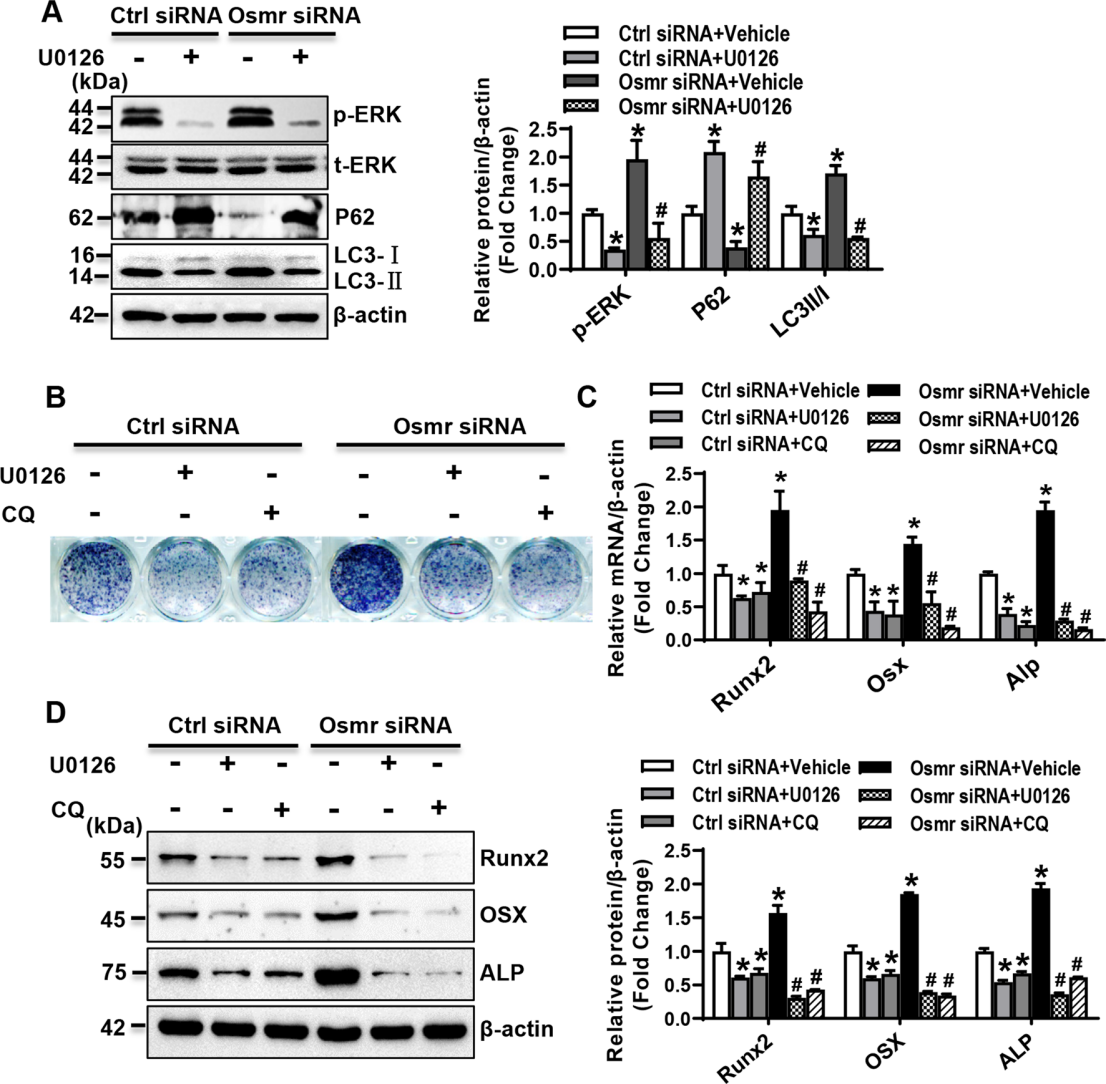
OSMRregulated osteogenic differentiation via ERK/autophagy signaling. ST2 cells were transfected with Osmr siRNA or control siRNA, followed by U0126 or vehicle treatment for 24 h. The levels of the proteins indicating the activation of ERK and autophagy were examined (**A**). Osmr loss-of-function experiments were performed in ST2 cells treated with U0126 or chloroquine. ALP staining of differentiated osteoblasts was done 14 days after osteogenic treatment (**B**). The mRNA (**C**) and protein (**D**) levels of osteogenic factors were examined 72 h after osteogenic treatment. Values are mean ± SD (*n* = 3). *p < 0.05 *vs.* Ctrl siRNA plus Vehicle; ^#^*p* < 0.05 *vs.* Osmr siRNA plus Vehicle

To further investigate whether ERK/autophagy signaling mediates OSMR regulation of osteogenic differentiation, OSMR loss-of-function experiments were performed under the background of U0126 or chloroquine treatment in ST2 cells. We demonstrated that the stimulatory effect of Osmr siRNA on ERK/autophagy signaling was remarkably compromised in the presence of U0126 (Fig. 5A). Meanwhile, the stimulatory effect of Osmr siRNA on osteogenic differentiation was attenuated in the presence of U0126 or chloroquine, as evidenced by the blunted ALP staining (Fig. 5B) and the decreased levels of osteogenic factors in the cells treated with Osmr siRNA and U0126 or chloroquine *vs.* the cells treated with Osmr siRNA and vehicle (Fig. 5C, D).

### OSMR silencing relieved the bone loss phenotype in OVX mice

**Fig. 6:**
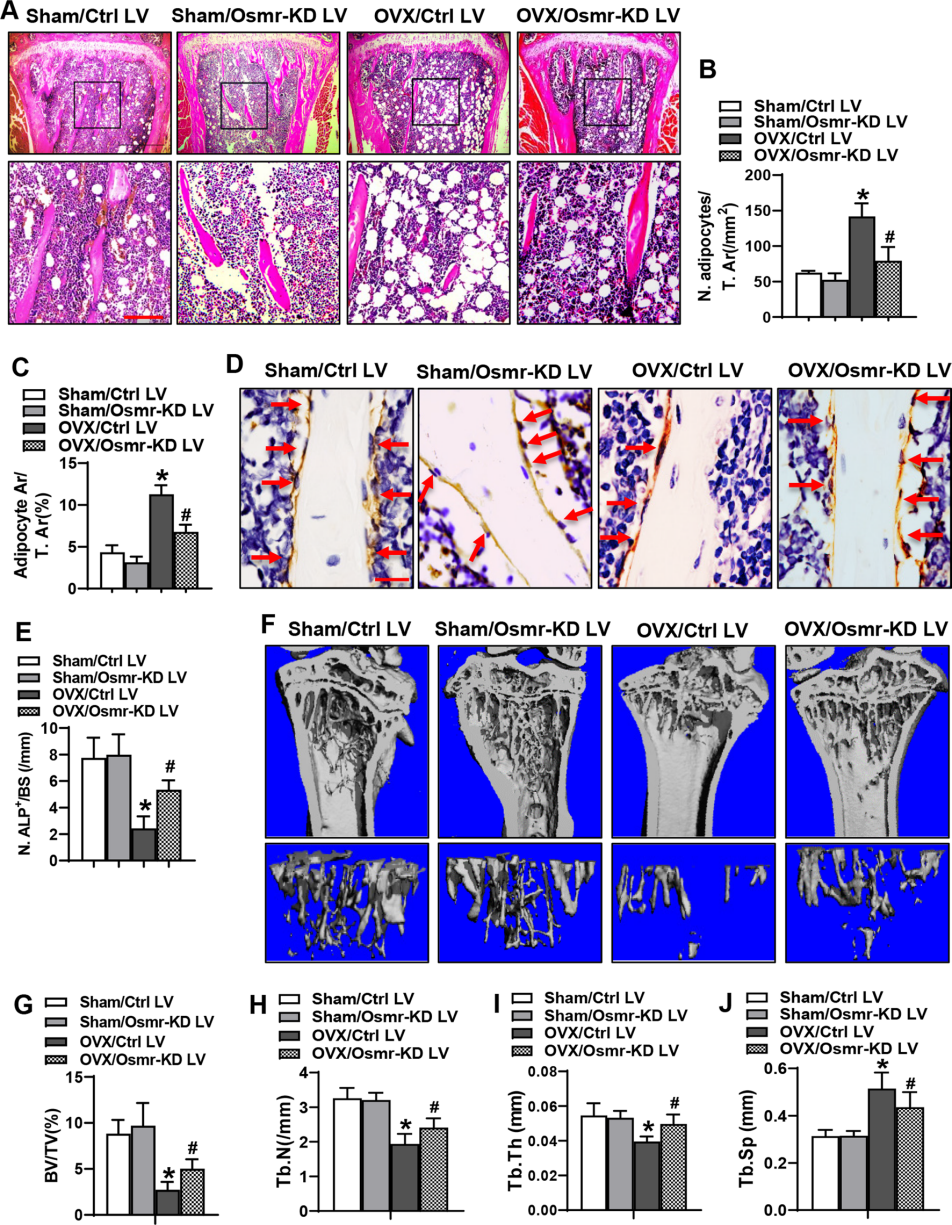
OSMR silencing relieved the bone loss phenotype in OVX mice. Representative images of H&E staining are shown. Scale bar, upper panel: 500 μm; lower panel: 100 μm (**A**). The number (**B**) and area (**C**) of adipocytes were quantified. Representative images of ALP immunohistochemical staining are shown. Scale bar: 25 μm (**D**). The number of osteoblasts on the trabeculae was quantified (**E**). μCT analysis of bone mass in the proximal metaphysis of tibiae was done and the reconstruction images are shown (**F**). Histomorphometric parameters were analyzed in the mice (**G**–**J**). Values are mean ± SD. **A**–**E,** *n* = 5; **G**–**J**, *n* = 8. *p < 0.05 vs. Sham/Ctrl LV. ^#^*p* < 0.05 *vs.* OVX/Ctrl LV

We further explored if OSMR plays a role in the differentiation of progenitor cells and even in bone homeostasis in vivo. OVX and Sham mice were transplanted with BMSCs infected with either Osmr-KD LV or control LV, respectively. Histological analysis of the tibiae in the mice 4 weeks after surgery revealed that adipocytes in the marrow were more (Fig. 6A–C), and ALP-positive osteoblasts on the trabeculae were fewer (Fig. 6D, E) in “OVX/Ctrl LV” mice than in “Sham/Ctrl LV” mice. Transplantation of Osmr-silenced BMSCs to sham mice did not significantly alter marrow adipocyte number and adipocyte area, as well as ALP-positive osteoblasts (Fig. 6A–C). By contrast, the number and area of adipocytes in “OVX/Osmr-KD LV” mice were reduced (Fig. 6A–C) and the number of ALP-positive osteoblasts was increased (Fig. 6D, E) in “OVX/Osmr-KD LV group as compared to the “OVX/Ctrl LV” group. Moreover, 12 weeks after surgery, μCT analysis revealed that when compared to “Sham/Ctrl LV” mice, “OVX/Ctrl LV” mice showed a bone loss phenotype, as revealed by the decrease in bone volume percentage (BV/TV, 69% decrease), trabecular number (Tb.N, 40% decrease), trabecular thickness (Tb.Th, 27% decrease), and the increase in trabecular space (Tb.Sp, 64% increase). However, these parameters in “OVX/Ctrl LV” mice were ameliorated after transplantation of Osmr-silenced BMSCs in “OVX/Osmr-KD LV” mice (Fig. 6F–J). Briefly, BV/TV, Tb.N, and Tb.Th were increased by 83%, 24%, and 26%, respectively, and Tb.Sp was decreased by 15% in “OVX/Osmr-KD LV” group *vs.* “OVX/Ctrl LV” group.

### OSMR in stromal progenitor cells regulated osteoclast differentiation

To explore whether OSMR in osteoblastic lineage regulates osteoclast formation, we investigated the correlation of RANKL and OPG to OSMR expression. The data showed that the mRNA and protein levels of RANKL were decreased and those of OPG were increased in the BMSCs underexpressing Osmr (Additional file 1: Fig. S7A-C). When cocultured with bone marrow osteoclast precursors, the BMSCs underexpressing Osmr largely inhibited osteoclast formation and downregulated the mRNA and/or protein expression levels of osteoclastogenic factors including NFATC1, CTSK, and TRAP (Additional file 1: Fig. S7D-G).

**Fig. 7:**
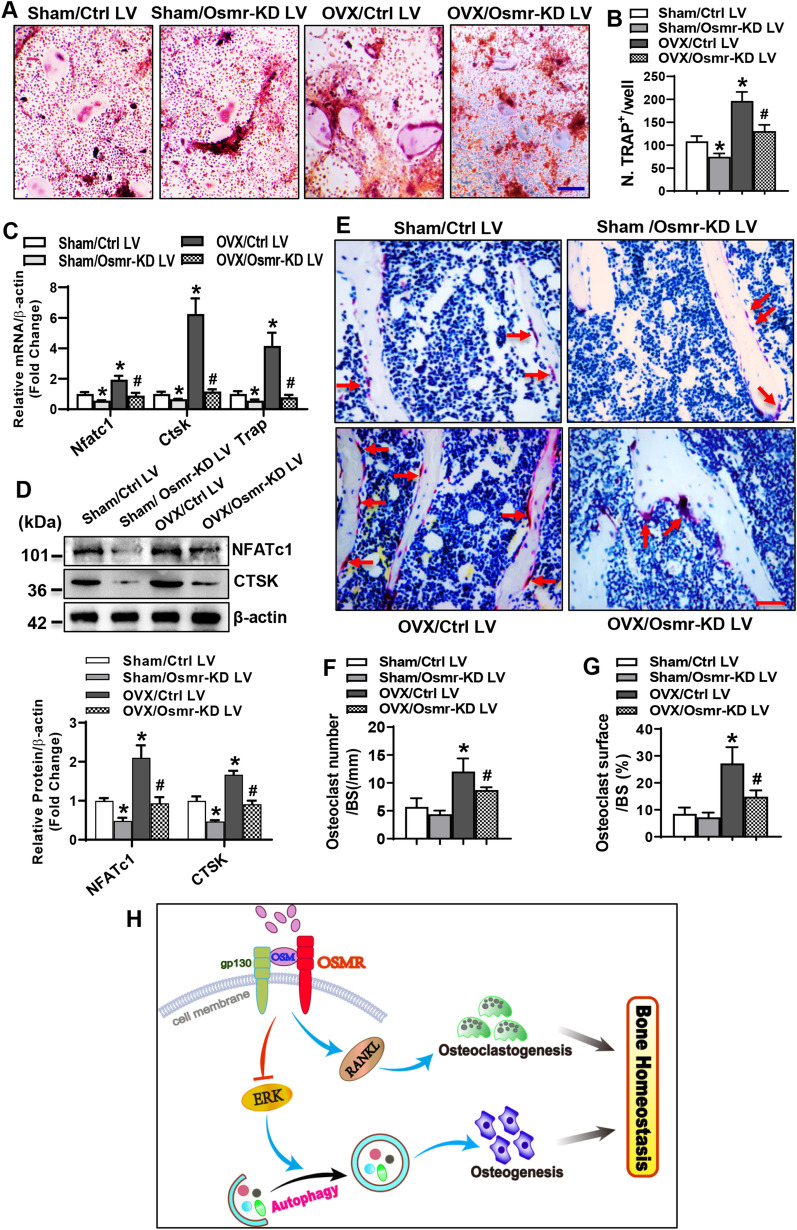
OSMR silencing attenuated osteoclast differentiation in ovariectomized mice. Bone marrow cells were isolated from the transplanted mice, cultured, and induced to allow osteoclast differentiation. TRAP staining was performed (**A**), osteoclasts were counted (**B**), and the mRNA and/or protein expression levels of osteoclastogenic factors were examined (**C**, **D**)**.** Scale bar in (**A**): 200 μm. TRAP staining was performed on the transplanted tibia sections and representative images are shown (**E**). Scale bar: 50 μm. The number (**F**) and surface (**G**) of the osteoclasts on the trabeculae were quantified. Schematic diagram depicting the mechanism for OSMR in regulating osteoblast and osteoclast differentiation is shown (**H**). Values are mean ± SD. **B,** *n* = 4; **C**, **D**, *n* = 3; **E**, **F**, *n* = 5. *p < 0.05 *vs.* Sham/Ctrl LV. ^#^*p* < 0.05 *vs.* OVX/Ctrl LV

Furthermore, 2 weeks after surgery, the tibial marrow cells from “OVX/Ctrl LV” mice formed more osteoclasts and expressed higher levels of osteoclastogenic genes, while those from “Sham/Osmr-KD LV” group formed fewer osteoclasts and expressed lower levels of osteoclastogenic genes than those from “Sham/Ctrl LV” group when induced for differentiation (Fig. 7A–D). However, the induction of osteoclastogenesis in “OVX/Ctrl LV” mice was attenuated in the tibial marrow cells from “OVX/Osmr-KD LV” mice, as evidenced by the reduction in osteoclast number and mRNA and/or protein expression levels of osteoclastogenic genes (Fig. 7A–D).

Moreover, TRAP staining of the tibiae in the mice 4 weeks after surgery revealed that osteoclast number and surface on the trabeculae were more in “OVX/Ctrl LV” mice than in “Sham/Ctrl LV” mice. Transplantation of Osmr-silenced BMSCs to sham mice did not significantly alter osteoclast number and osteoclast surface (Fig. 7E–G). By contrast, the number and surface of osteoclasts in “OVX/Osmr-KD LV” mice were reduced as compared to the “OVX/Ctrl LV” group (Fig. 7E–G).

## Discussion

OSMR and LIFR are the two receptors known to mediate the action of OSM ligand. Of interest, the two receptors transduce distinct functions of OSM in stromal and osteoblastic cells. When acting through LIFR, OSM inhibited sclerostin production and stimulated bone formation [21, 37]. In contrast, when acting through OSMR, OSM stimulated RANKL production and induced osteoclast differentiation [21]. Mice lacking OSMR were characterized by an osteopetrotic phenotype due to an impaired osteoclast formation [21]. Moreover, the Osmr null mice had low number of osteoblasts and low osteoid, whereas MAR was not significantly altered in the tibiae. The calvarial osteoblasts from the Osmr null mice had impaired ALP activity and mineralized nodule formation, whereas the expression levels of osteogenic factors such as Runx2, osterix, and ALP were not changed [21]. Given these discrepancies, the direct regulatory role of OSMR in osteoblast differentiation needs to be further validated.

In this study, Osmr expression was detected in various tissues of mice. It was highly expressed in bone and fat. In addition, we provided evidence that OSMR expression was altered in BMSCs after osteogenic or adipogenic treatment. It increased at almost all time points throughout osteogenic differentiation. In contrast, it increased at early stage of adipogenic differentiation while decreased at late stage. Furthermore, Osmr expression was induced in the radial metaphysis of ovariectomized or aged mice. These data raised the possibility that OSMR may have a direct role in the differentiation of BMSCs and is implicated in age-related or postmenopausal bone loss.

To verify our hypothesis, gain-of-function and loss-of-function studies were conducted in marrow stromal progenitor cells. The results showed that OSMR suppressed osteogenesis and conversely stimulated adipogenesis. These findings are surprising because they are not consistent to the findings in Walker et al.’ s report in which they revealed fewer osteoblasts and less osteoid despite unchanged MAR in the tibiae of Osmr KO mice [21]. The major defect of the Osmr KO mice was the impaired bone resorption that dominated over the impaired bone formation, leading to the increase in bone mass. It is known that, in ovariectomized rats and mice which mimic the situation of postmenopausal women, the osteoblast number and osteoid surfaces were increased along with the increase in osteoclast numbers and surfaces, especially at the relatively early stage after surgery [38–41]. Likely, there might be the possibility that the reduction in osteoblasts took place in the Osmr KO mice as a tight coupling response to the impaired osteoclastic bone resorption. Another possibility is that the reduction in osteoblasts was attributed to some other molecule(s) released by cells other than osteoblasts due to the deletion of Osmr in the cells. The inhibitory effect of OSMR on osteogenesis that we found in the current study may represent its direct regulatory function in the differentiation of stromal progenitor cells.

Once binding to its ligand, OSMR forms a co-receptor complex with gp130 receptor. Thereafter, Janus tyrosine kinases (JAKs) are activated and in turn phosphorylate several cytoplasmic tyrosine residues in the gp130:OSMR complex that provide the docking sites for SH2 domain-containing molecules such as signal transducers and activators of transcription (STATs), and the tyrosine phosphatase SHP-2 or the adaptor protein SHC1. Once recruited to the receptor, SHP-2 or SHC1 becomes phosphorylated and recruits further adaptor proteins to activate Ras/Raf/MEK/ERK, or phosphatidylinositol-3-kinase (PI3K)/AKT pathway [18, 42, 43]. Of interest, in the current study, by using gain-of-function and loss-of-function experiments, we have demonstrated that OSMR downregulated the level of p-SHC1 and suppressed ERK signaling in the presence of osteogenic medium. These data suggest that OSMR may have distinct effects on ERK signaling in different cell types and under different culture conditions.

Autophagy has recently been identified as a necessary part in differentiation of osteoblasts and bone homeostasis. In vitro silencing of Beclin-1 or ATG7 in osteoblastic cells impaired mineralization [12]. Furthermore, targeted deletion of autophagy regulators such as FAK family-interacting protein of 200 kDa (Fip200), ATG5 or ATG7 in osteoblast linages impaired terminal differentiation of osteoblasts and bone formation and/or mineralization, leading to reduction in bone mass [12, 44–46]. A variety of signaling pathways are known to regulate autophagy including PI3K-Akt-mTOR pathway, ERK, and c-Jun N-terminal kinase (JNK) pathways [47]. ERK signaling stimulates autophagy via phosphorylating Gα-interacting protein (GAIP) that accelerates the GTPase activity of Gαi3 protein and ultimately promotes autophagy [48].

In this study, using gain-of-function and loss-of-function experiments, we demonstrated that OSMR decreased the level of autophagy-related factors and inhibited LC3 puncta formation in stromal progenitor cells, indicating the involvement of autophagy in OSMR-regulated osteogenesis. Further in-depth mechanistic investigation showed that the inhibition of ERK1/2 signaling downregulated the key autophagy regulators and alleviated osteogenesis, and this inhibitory effect on osteogenesis was recapitulated by the treatment with autophagy inhibitor. Furthermore, the osteoblast differentiation induced by Osmr siRNA was compromised in the cells with the inactivation of either ERK or autophagy. These clearly certified that OSMR downregulates osteogenesis through inhibiting ERK-mediated autophagy signaling.

Of interest, opposite to the inhibitory effect of OSMR on autophagy that we herein found, Hu et al. previously reported that OSM, through OSMR, upregulated cardiomyocyte autophagy in infarcted heart via suppressing mammalian Ste20-like kinase 1 [49], suggesting that OSMR may either stimulate or inhibit autophagy in different types of cells. A possible explanation for this ambivalence is that OSMR is able to activate both pro-autophagic and anti-autophagic signaling pathways, depending on cellular context. A similar dual role in the regulation of autophagy was also found for Ras, which was shown to either activate or inactivate autophagy through several signaling pathways, leading to either tumor progression or repression [50].

By now what interests us most is whether the bone homeostasis could be improved if we alter the expression of OSMR in marrow stromal progenitor cells. The bone loss observed in the ovariectomized mice was ameliorated after tibial transplantation of Osmr-silenced BMSCs. Consistent to the in vitro findings, osteoblasts were more and adipocytes were fewer in these mice than in the ovariectomized mice transplanted with control BMSCs. Moreover, as expected, the enhanced osteoclast formation was ameliorated in the ovariectomized mice after transplantation of Osmr-silenced BMSCs. In support of this, we demonstrated that the depletion of Osmr in BMSCs decreased RANKL and increased OPG expression, thereby suppressed the formation of osteoclasts when cocultured with marrow osteoclast precursors. This is in line with Walker’s report that OSMR facilitated osteoclast formation and bone resorption via increasing RANKL expression [21].

Therefore, combined with previous published reports, our findings suggest that OSMR may negatively regulate bone homeostasis through controlling both osteoblastic bone formation and osteoclastic bone resorption (Fig. 7H). This study was performed on cells and mice, whether OSMR works the same way in human needs further investigation.

## Conclusions

In summary, we have established the direct role of OSMR in regulating osteogenic differentiation of marrow stromal progenitor cells*.* The underlying mechanism involves the inhibition of autophagy mediated by the inactivation of ERK1/2 signaling pathway. The silencing of OSMR in marrow stromal cells benefits bone homeostasis through increasing osteoblasts and decreasing osteoclasts. Our study helps to uncover a new therapeutic target for metabolic bone disorders such as osteoporosis.

## Supplementary Information

**Additional file 1.** Supplementary Figures and Tables.

## Data Availability

The data that support the findings of this study are available from the corresponding author upon reasonable request.

## Abbreviations

ALP: Alkaline phosphatase
ATG: Autophagy-related gene
BMP: Bone morphogenetic protein
BMSC: Bone marrow stem cell
BSP: Bone sialoprotein
C/EBP: CCAAT/enhancer-binding protein
CTSK: Cathepsin K
ERK: Extracellular signal-regulated kinase
FABP4: Fatty acid-binding protein 4
LC3: Microtubule-associated protein 1 light chain 3
NFATC1: Nuclear factor of activated T cells 1
OPN: Osteopontin
OSM: Oncostatin M
OSMR: Oncostatin M receptor
OSX: Osterix
OVX: Ovariectomy
PPARγ: Peroxisome proliferator-activated receptor γ
Runx2: Runt-related transcription factor 2
RANKL: Receptor activator of NF-kB ligand
SHC1: Src homology 2 domain-containing transforming protein C1
TRAP: Tartrate-resistant acid phosphatase
ULK: Unc-51 like kinase 1
