# Supplementary material for: Oncostatin M receptor regulates osteoblast differentiation via extracellular signal-regulated kinase/autophagy signaling
Source: Stem Cell Res Ther. 2022 Jun 28;13:278. doi: 10.1186/s13287-022-02958-1 (PMC9241272; doi:10.1186/s13287-022-02958-1)
Supplement: Supplementary file 1 — Additional file 1. Supplementary Figures and Tables. [file 13287_2022_2958_MOESM1_ESM.pdf]

Figure S1

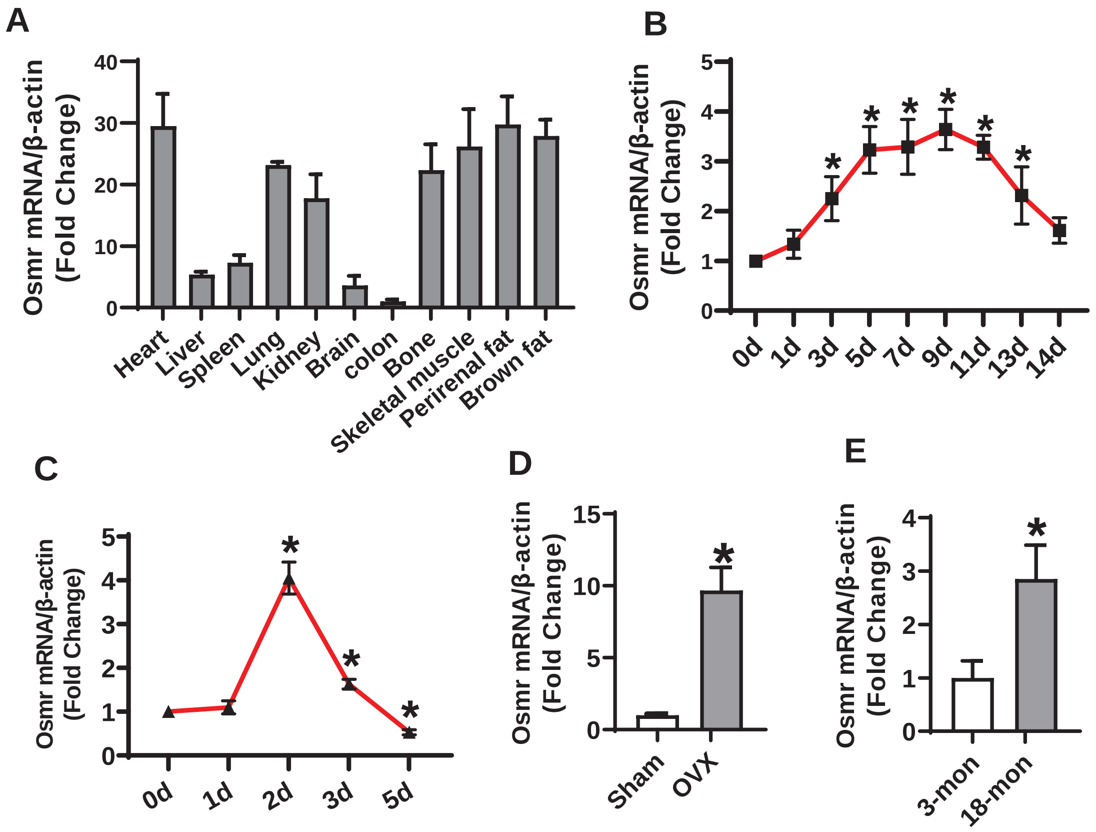

Fig. S1. Osmr was expressed in bone and regulated during osteogenic and adipogenic differentiation. Osmr level was detected in various tissues of mice by qRT-PCR. Osmr level in colon was set to 1 (A). Osmr level was detected in BMSCs cultured in osteogenic (B) or adipogenic (C) medium by qRT-PCR. Osmr level at d 0 was set to 1. Osmr expression was examined in the radial metaphysis of OVX mice (D) or aged mice (E) using qRT-PCR. Values are mean ± SD (n=3). *$p < 0.05$ *vs*. day 0 (B, C), or Sham (D) or 3-month-old mice (E).

Figure S2

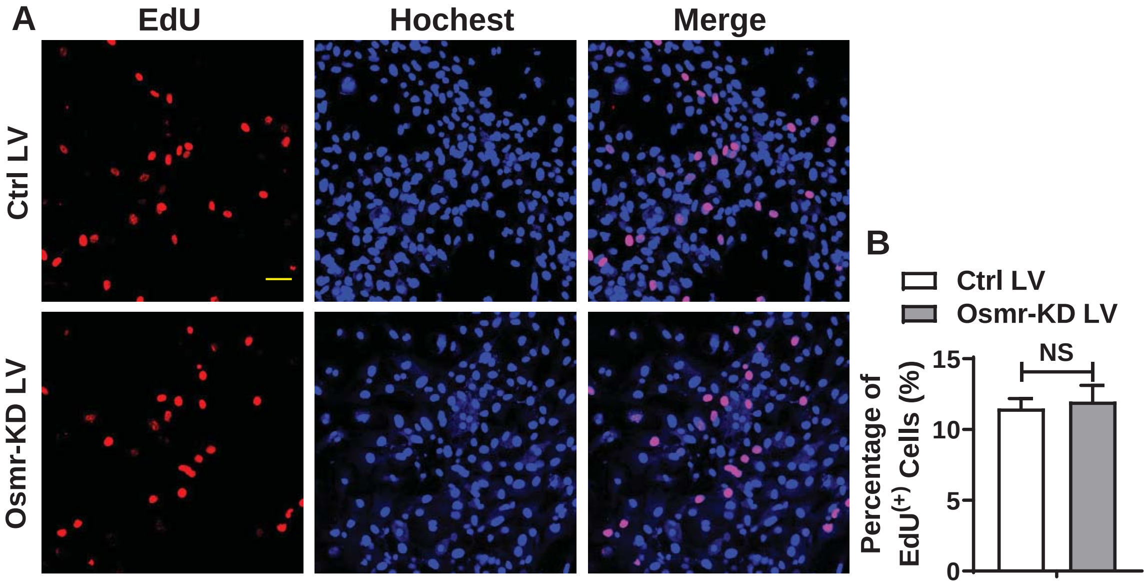

Fig. S2. The effect of Osmr silencing on proliferation of primary BMSCs. EdU staining was performed to evaluate the effect of Osmr silencing on cell proliferation of primary BMSCs 48 h after infection with Osmr-KD LV or control LV **(A)**. The percentage of EdU-positive cells was analysed by image J software **(B)**. Scale bar: 50 μm. Values are mean ± SD (n=3). *p < 0.05 *vs*. Ctrl LV.

Figure S3

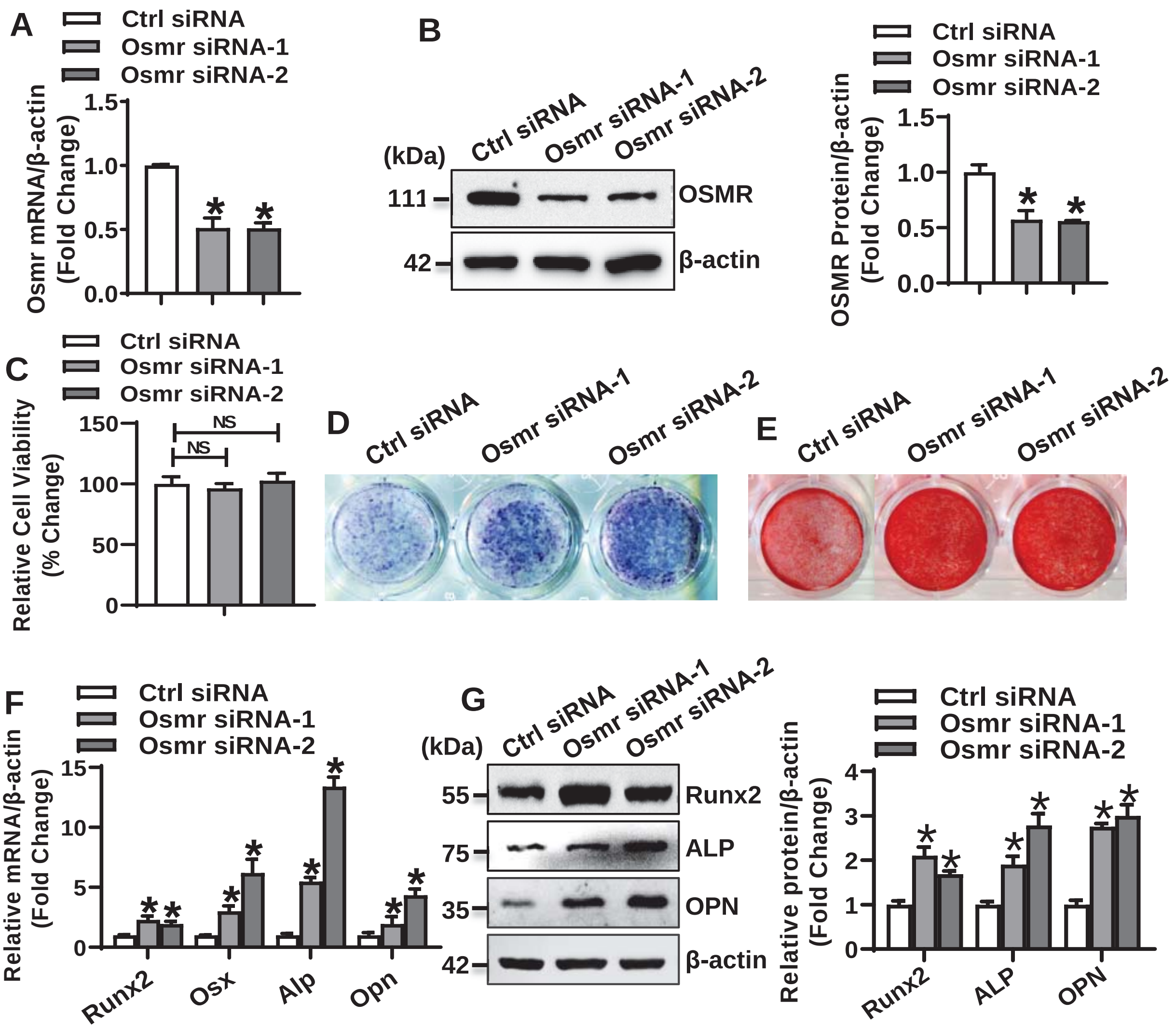

Fig. S3. Silencing of OSMR in ST2 cells enhanced osteogenesis. ST2 cells were transfected with Osmr siRNAs or control siRNA. Silencing of OSMR in ST2 was detected by using qRT-PCR **(A)** and Western blotting **(B)**. The effect of Osmr siRNAs on the viability of ST2 cells was detected by CCK-8 assay **(C)**. After transfection, the cells were induced with osteogenic medium to allow differentiation. ALP staining **(D)** and alizarin red staining **(E)** of differentiated osteoblasts were done 14 days and 21 days, respectively, after osteogenic treatment. The mRNA **(F)** and protein **(G)** levels of osteogenic factors were examined 72 h after osteogenic treatment. Values are mean ± SD. (A, B, F, G), n=3. (C), n = 10. *p < 0.05 *vs*. Ctrl siRNA.

Figure S4

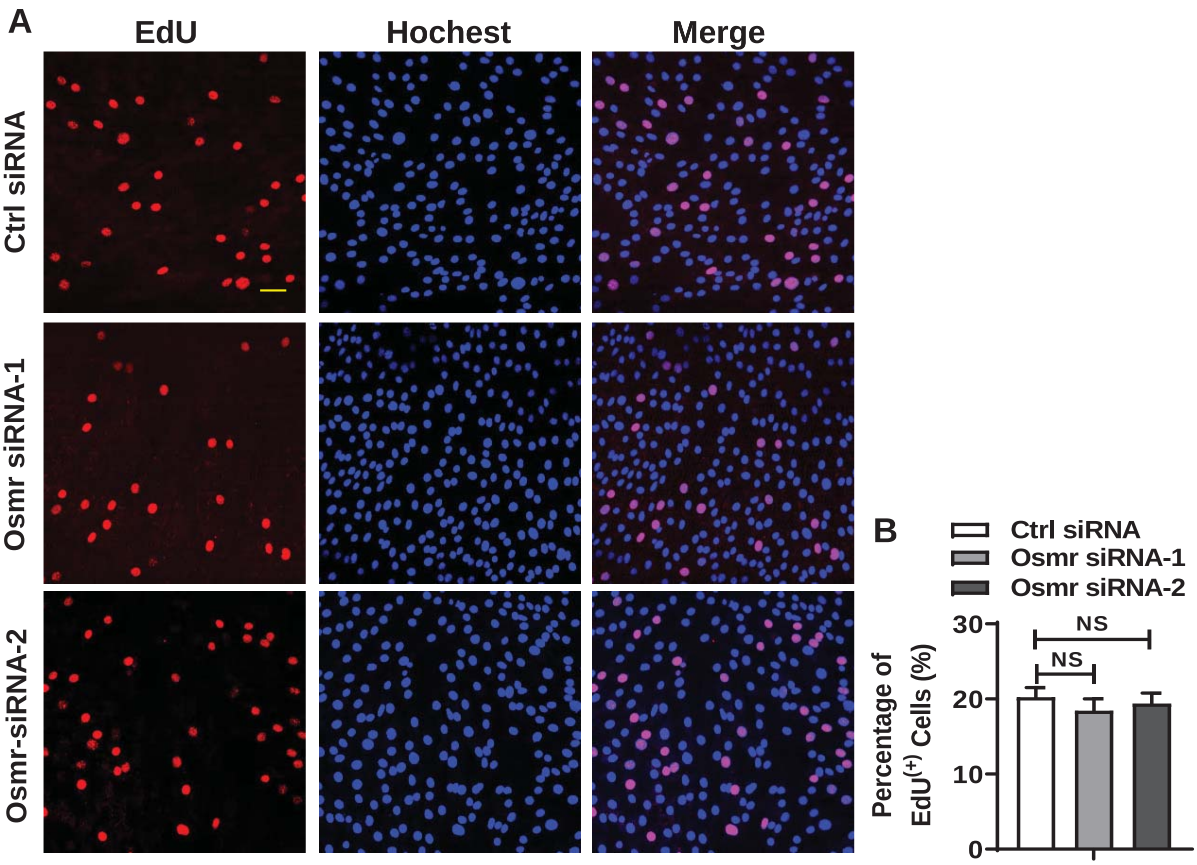

Fig. S4. The effect of Osmr silencing on proliferation of ST2 cells. EdU staining was performed to evaluate the effect of Osmr silencing on proliferation of ST2 cells 48 h after transfection with Osmr siRNAs or control siRNA **(A)**. The percentage of EdU-positive cells was analysed by image J software **(B)**. Scale bar: 50 μm. Values are mean ± SD (n=3). *$p < 0.05$ *vs*. Ctrl siRNA.

Figure S5

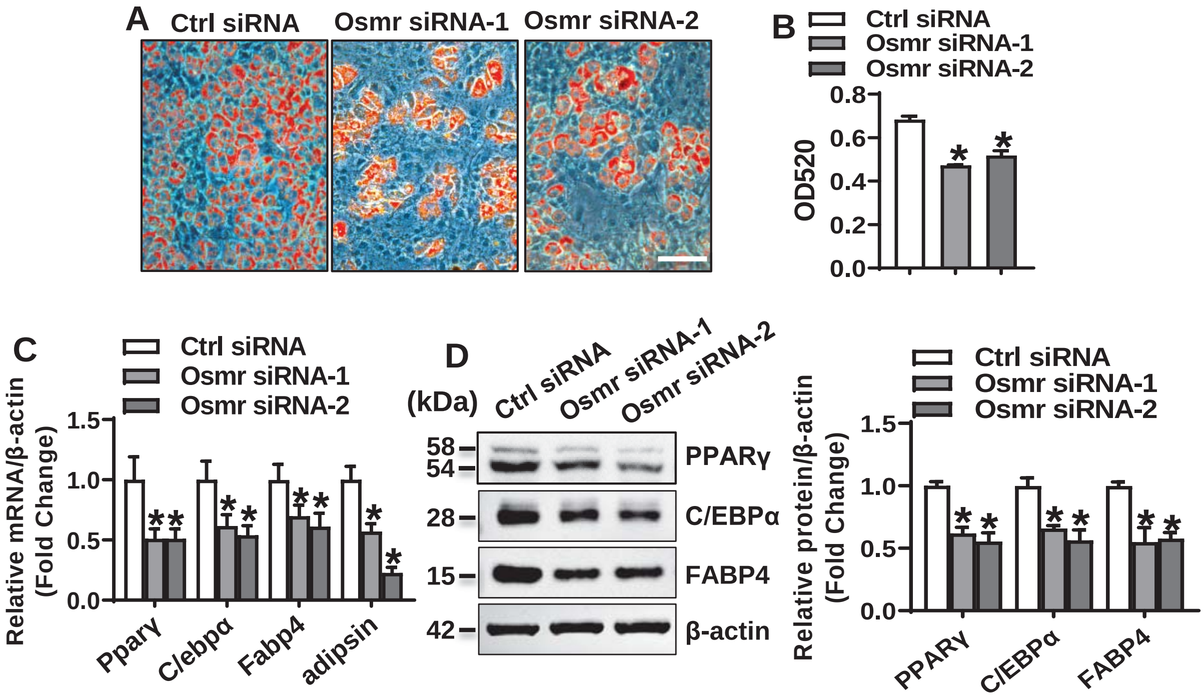

Fig. S5. Silencing of OSMR in ST2 cells suppressed adipogenesis. ST2 cells were transfected with Osmr siRNAs or control siRNA, then induced with adipogenic medium to allow differentiation. Lipid droplet formation in differentiated adipocytes was detected by staining with oil-red O 5 days after induction **(A)**. Oil-red O stain was extracted and absorbance was measured by spectrophotometry at 520 nm **(B)**. The mRNA **(C)** and protein **(D)** levels of adipogenic factors were examined 48 h and 72 h, respectively, after induction. Scale Bar in (A): 50 μm. Values are mean ± SD, n=3. *p < 0.05 *vs*. Ctrl siRNA.

Figure S6

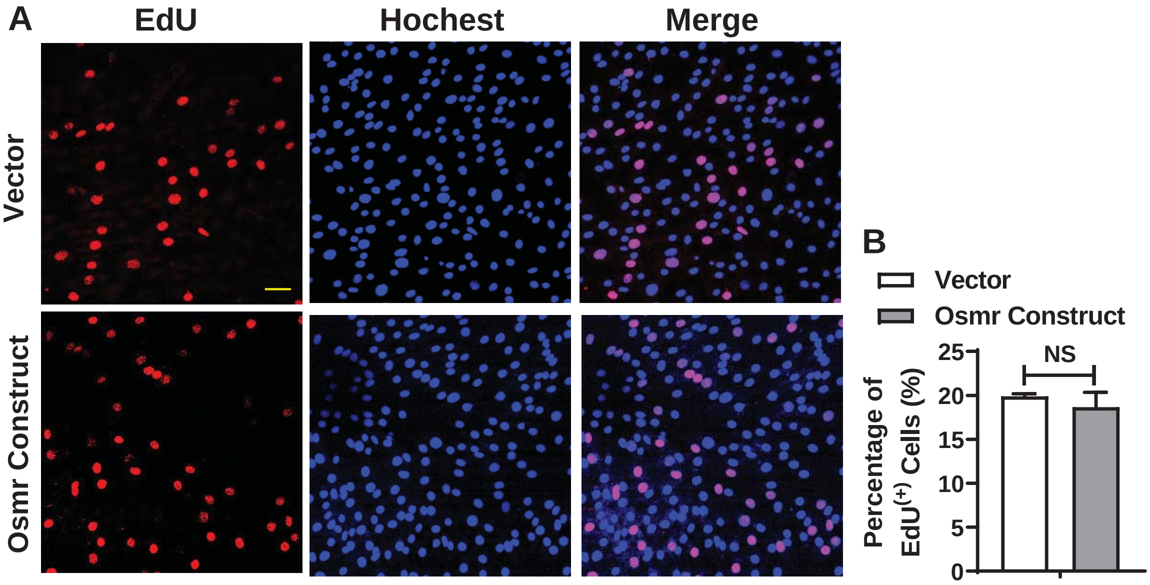

Fig. S6. The effect of Osmr overexpression on proliferation of ST2 cells. EdU staining was performed to evaluate the effect of Osmr overexpression on proliferation of ST2 cells 48 h after transfection with Osmr construct or vector **(A)**. The percentage of EdU-positive cells was analysed by image J software **(B)**. Scale bar: 50 μm. Values are mean ± SD (n=3). *$p < 0.05$ *vs*. Vector.

Figure S7

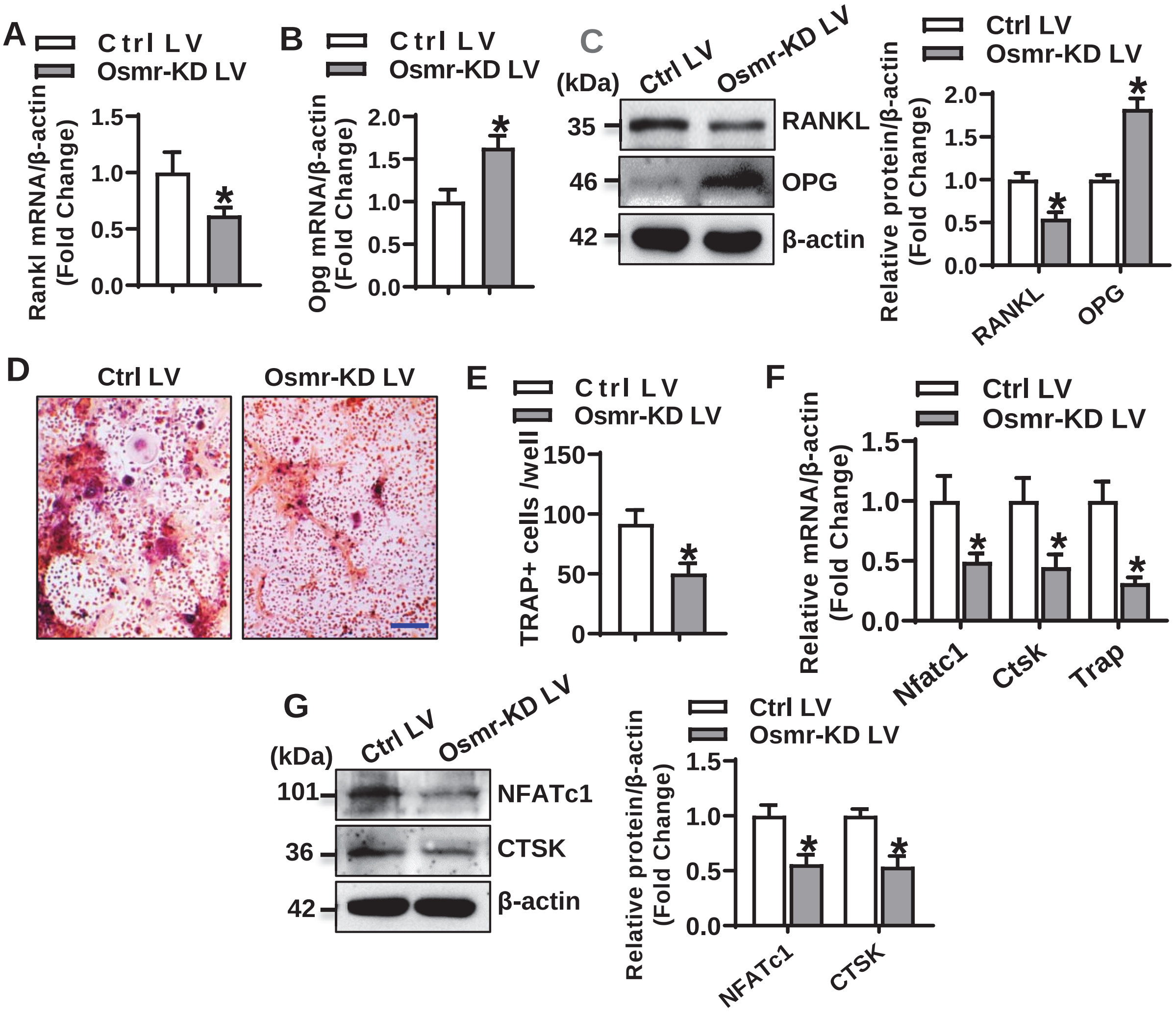

Fig. S7. OSMR in stromal progenitor cells regulated osteoclast differentiation. Primary BMSCs were infected with Osmr-KD lentivirus or control virus. The mRNA and protein levels of RANKL and OPG were examined in the cells 48 h after transfection **(A-C)**. Wild-type bone marrow cells were cocultured with control or Osmr-silenced BMSCs, and induced for osteoclast differentiation. TRAP staining was performed 7 days after induction **(D)**, osteoclasts were counted **(E)** and the mRNA and/or protein levels of osteoclastogenic factors were examined 5 days after induction **(F, G)**. Scale bar in (D): 200 μm. Values are mean ± SD (n=3). *p < 0.05 *vs*. Ctrl LV.

Supplemental Table 1. Primers used for qRT-PCR

Gene Names	Forward primer sequences	Reverse primer sequences
Runx2	CCTGAACTCTGCACCAAGTCCT	TCATCTGGCTCAGATAGGAGGG
Osx	GGCTTTTCTGCGGCAAGAGGTT	CGCTGATGTTTGCTCAAGTGGTC
Bglap	GCAATAAGGTAGTGAACAGACTCC	CCATAGATGCGTTTGTAGGCGG
Alp	TCTTGTCCGTGTCGCTCACCAT	CCAGAAAGACACCTTGACTGTGG
Opn	GCTTGGCTTATGGACTGAGGTC	CCTTAGACTCACCGCTCTTCATG
PPARγ	CTTGACAGGAAAGACAACGG	GCTTCTACGGATCGAAACTG
C/EBPα	CTGATTCTTGCCAAACTGAG	GAGGAAGCTAAGACCCACTAC
Fabp4	AAATCACCGCAGACGACAGG	GGCTCATGCCCTTTCATAAAC
adipsin	TGATGTGTGCAGAGAGCAAC	CGTAACCACACCTTCGACTG
Osmr	CCACTTCTGGAAATGAGCGAC	ATGCTGCTTCCATTCTCCGACC
Nfatc1	GGTGCCTTTTGCGAGCAGTATC	CGTATGGACCAGAATGTGACGG
Ctsk	AGCAGAACGGAGGCATTGACTC	CCCTCTGCATTTAGCTGCCTTTG
Trap	GCGACCATTGTTAGCCACATACG	CGTTGATGTCGCACAGAGGGAT
Lifr	CTTCGATCCTCAACACAGAGCAG	CGCTTGCTCTACTGTGATGTCG
β-actin	AAGACCTCTATGCCAACACAG	GGAGGAGCAATGATCTTGATC

Supplemental Table 2. siRNAs used for gene silencing

Names	sense（5'-3'）	antisense（5'-3'）
Osmr siRNA-1	GGAAGAACCUUGGUCCCAATT	UUGGGACCAAGGUUCUUCCTT
Osmr siRNA-2	GCAGCAUUCUGUCACUCAUTT	AUGAGUGACAGAAUGCUGCTT
